# Lysosomal cathepsins act in concert with Gasdermin-D during NAIP/NLRC4-dependent IL-1β secretion

**DOI:** 10.1038/s41419-022-05476-3

**Published:** 2022-12-08

**Authors:** Laura Migliari Branco, Marcelo Pires Amaral, Henning Boekhoff, Ana Beatriz Figueiredo de Lima, Ingrid Sancho Farias, Silvia Lucena Lage, Gustavo José Silva Pereira, Bernardo Simões Franklin, Karina Ramalho Bortoluci

**Affiliations:** 1 Departamento de Farmacologia, Escola Paulista de Medicina, Universidade Federal de São Paulo (UNIFESP), São Paulo, Brazil; 2 National Institute of Allergy and Infectious Diseases, National Institute of Health, Bethesda, USA; 3 Institute of Innate Immunity, University Hospitals, Bonn, Germany; 4 Present Address: Division of Functional Genome Analysis, German Cancer Research Center (DKFZ), Heidelberg, Germany

**Keywords:** Inflammasome, NOD-like receptors

## Abstract

The NAIP/NLRC4 inflammasome is classically associated with the detection of bacterial invasion to the cytosol. However, recent studies have demonstrated that NAIP/NLRC4 is also activated in non-bacterial infections, and in sterile inflammation. Moreover, in addition to the well-established model for the detection of bacterial proteins by NAIP proteins, the participation of other cytosolic pathways in the regulation of NAIP/NLRC4-mediated responses has been reported in distinct contexts. Using pharmacological inhibition and genetic deletion, we demonstrate here that cathepsins, well known for their involvement in NLRP3 activation, also regulate NAIP/NLRC4 responses to cytosolic flagellin in murine and human macrophages. In contrast to that observed for NLRP3 agonists, cathepsins inhibition did not reduce ASC speck formation or caspase-1 maturation in response to flagellin, ruling out their participation in the effector phase of NAIP/NLRC4 activation. Moreover, cathepsins had no impact on NF-κB-mediated priming of pro-IL-1β, thus suggesting these proteases act downstream of the NAIP/NLRC4 inflammasome activation. IL-1β levels secreted in response to flagellin were reduced in the absence of either cathepsins or Gasdermin-D (GSDMD), a molecule involved in the induction of pyroptosis and cytokines release. Notably, IL-1β secretion was abrogated in the absence of both GSDMD and cathepsins, demonstrating their non-redundant roles for the optimal IL-1β release in response to cytosolic flagellin. Given the central role of NAIP/NLRC4 inflammasomes in controlling infection and, also, induction of inflammatory pathologies, many efforts have been made to uncover novel molecules involved in their regulation. Thus, our findings bring together a relevant contribution by describing the role of cathepsins as players in the NAIP/NLRC4-mediated responses.

## Introduction

Inflammasomes are multiprotein platforms composed of a sensor protein of the nucleotide-binding oligomerization domain (NBD), leucine-rich repeat (LRR)-containing protein (NLR) or the pyrin and, HIN domain-containing protein (PYHIN) families, that are assembled after a diverse range of stimuli, including pathogen- and host-derived signals [1, 2]. Inflammasome assembly drives the recruitment and activation of caspase-1, resulting in the processing and release of the mature forms of the interleukins (IL)-1β and IL-18 and in the induction of a highly pro-inflammatory cell death termed pyroptosis [3].

NAIP/NLRC4 inflammasome is one of the best-characterized inflammasomes and has an important role in the restriction of intracellular pathogenic bacteria, such as *Salmonella typhimurium* [4, 5] and *Legionella pneumophila* [6]. Importantly, recent studies have demonstrated a role for NAIP/NLRC4 inflammasomes in response to non-bacterial infections [7–10] and sterile inflammation triggers [11–16]. However, the molecular mechanism of NAIP/NLRC4 activation under these conditions remains to be fully elucidated.

NAIP/NLRC4 inflammasome is classically described to assemble in the presence of specific bacterial proteins in the cytosol, such as flagellin, a subunit of bacterial flagella, and components of type III and IV bacterial secretion systems [17]. Members of the NAIP subfamily of proteins recognize bacterial components [18, 19], and recruit NLRC4 to assemble a NAIP/NLRC4 inflammasome [20]. In addition to the direct binding of bacterial ligands by NAIP proteins, other regulatory mechanisms participate in NAIP/NLRC4 activation under infections or sterile condition. These include post-translational modifications such as the NLRC4 serine 533 (S533) phosphorylation by Protein kinase C δ (PKCδ) [21, 22] or LRR kinase 2 (LRRK2) [23] and the interaction of NLRC4 with other inflammasomes members [13, 24, 25]. NLRC4 serine 533 (S533) phosphorylation by flagellin precedes its recognition by NAIP5 [22] and induces the optimal activation of NAIP5/NLRC4 [21, 22, 24]. Similarly, the recruitment of NLRP3 in response to *S. typhimurium* infection or flagellin stimulation also contributes to boost NAIP/NLRC4-mediated responses in murine and human cells [24–26]. Importantly, our group has previously demonstrated that the stimulation of macrophages with cytosolic flagellin triggers a lysosomal pathway that regulates NLRC4-dependent IL-1β secretion [27]. Nonetheless, the mechanism by which lysosomal pathway/cathepsins modulate NAIP/NLRC4 inflammasome responses remains obscure.

Lysosomal membrane permeabilization (LMP) induces the leakage of cathepsins to the cytosol, and is commonly associated with NLRP3 activation. Cathepsin B was the first proposed to be involved in NLRP3 activation based on its pharmacological inhibition with Ca-074Me, which was shown to reduce IL-1β production in response to particulate agonists [28]. Subsequent studies have implicated multiple cathepsins in NLRP3 activation. Because these proteases share similar enzymatic activities, it is possible that they play redundant roles in NLRP3 inflammasome signaling [29, 30]. Cathepsins participate in multiple steps of NLRP3 activation. More recent studies suggest that these proteases act not only in the assembly and effector phase of NLRP3 activation but also contribute to the priming stage, since treatment with different inhibitors, or combined knockdown of multiple cathepsins were able to reduce pro-IL-1β transcription [28]. Interestingly, cathepsin B was also shown to interact with NLRP3 at the endoplasmic reticulum leading to caspase-1 activation in response to the classical agonists such as nigericin, crystals or ATP [31]. Moreover, LMP seems to be a pathway that converge on potassium or calcium mobilization that trigger NLRP3 activation [32, 33].

In contrast to the well-established role in the regulation of NLRP3 inflammasomes, the participation of cathepsins in the activation of NAIP/NLRC4 inflammasomes remains to be clarified. Here, we demonstrated that the pharmacological or genetic inhibition of cathepsins reduced IL-1β secretion in response to flagellin, even in NLRP3-deficient macrophages, indicating that these proteases directly regulate NAIP5/NLRC4-mediated responses. Conversely to that found for NLRP3 agonists, cathepsins inhibition did not affect NF-κB-mediated responses required for the induction of pro-IL-1β and pro-caspase-1, nor interfered with ASC speck formation or caspase-1 cleavage in response to flagellin, demonstrating these proteases act downstream of NAIP/NLRC4 inflammasome assembly. Accordingly, cathepsins seem to act during secretion of IL-1β in response to flagellin. The lack of cathepsins or Gasdermin-D (GSDMD) partially inhibited the IL-1β secretion in response to the flagellin, whereas its secretion is aborted in the absence of both molecules. Together, our data demonstrate the cooperative role of GSDMD and cathepsins for NAIP/NLRC4-dependent- mature IL-1β secretion.

## Results

### Cathepsins regulate IL-1β secretion in response to flagellin

**Fig. 1:**
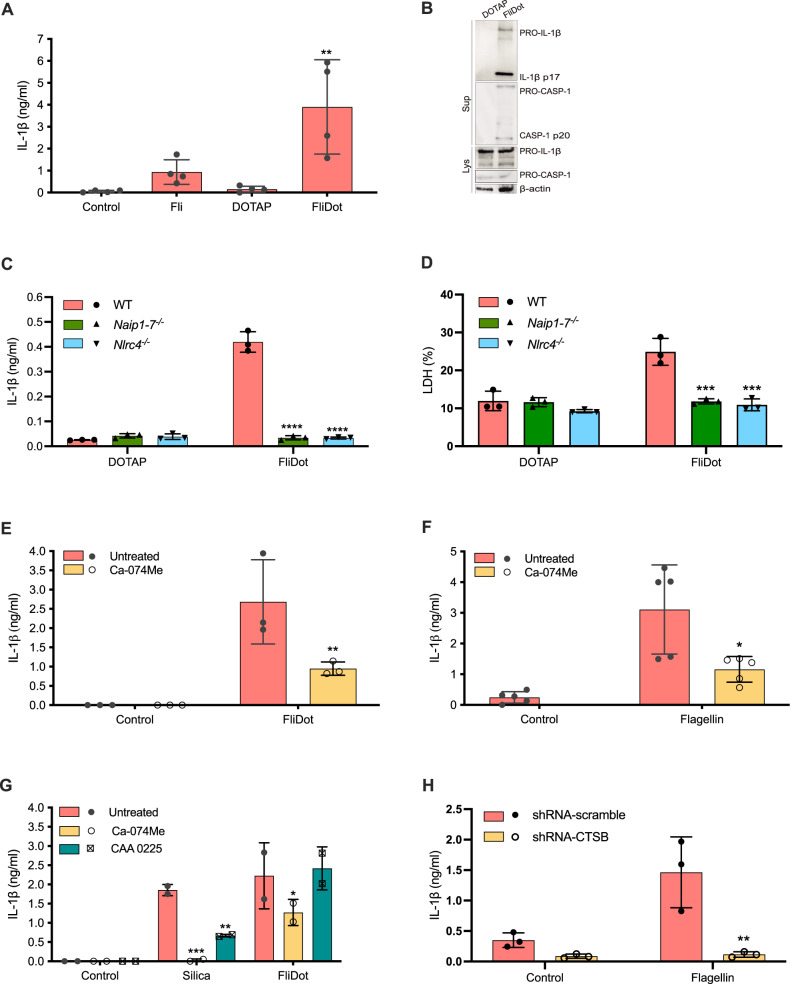
Cathepsins B plays a major role in the regulation of NAIP/NLRC4 dependent-responses to cytosolic flagellin. **A** Starch-elicited peritoneal macrophages (PMs) isolated from C57BL/6j wild-type (WT) mice were treated with LPS (500 ng/ml, 3 h) and stimulated with ultrapure flagellin extracted from *Salmonella typhimurium* in its free form (Fli) (1 μg/ml, 6 h), with empty DOTAP vesicles or with flagellin inserted into DOTAP (FliDot) (1 μg/ml, 6 h). IL-1β secretion was assessed in the culture supernatant by ELISA. The bars represent the average of four independent experiments performed in technical triplicates ± SD, **p < 0.01 (One-way ANOVA) when compared to cells treated with LPS alone or with LPS + DOTAP. **B** The secretion of the active forms of IL-1β and caspase-1 were detected by western blot (WB) of the cell culture supernatant. Pro-caspase-1, pro-IL-1β and β-actin were detected by WB in cell lysates (lys). Representative data from two independent experiments. **C**, **D** Bone marrow-derived macrophages (BMDM) from WT, *Naip1-7*^−/−^ and *Nlrc4*^*−/−*^ mice were treated with LPS and stimulated with empty DOTAP or FliDot. **C** IL-1β secretion was evaluated in the culture supernatant by ELISA. The numbers represent the mean ± SD of experimental triplicates. ****p < 0.0001 (Two-way ANOVA) when compared to WT cells. Data representative of three independent experiments. **D** Cytotoxicity was assessed by LDH release in the culture supernatant. The numbers represent the mean ± SD of experimental triplicates. ****p < 0.0001 (Two-way ANOVA) when compared to WT cells. Data representative of three independent experiments. **E** WT BMDMs were pretreated with the cathepsin B inhibitor Ca-074Me (25 μM) and primed with LPS. Next, the cells were stimulated with FliDot (1 μg/ml, 6 h). IL-1β release was evaluated in the culture supernatants by HTRF. The bars represent the average of three independent experiments performed in technical triplicates ± SD. **p < 0.01; ****p < 0.0001 (Two-way ANOVA) when compared to untreated cells. **F** PMA-differentiated (200 ng/mL, 24 h) THP-1 monocytic cells were transfected with ultrapure flagellin from *S. typhimurium* using Lipofectamine 3000 (1 μg/ml, 4 h) pretreated or not with cathepsin inhibitor Ca-074Me (25 μM). IL-1β secretion was assessed in the culture supernatant by ELISA. The bars represent quintuplicate of two independent experiments ± SD, *p < 0.05, ***p < 0.001 (One-way ANOVA) when compared to untreated cells. **G** WT BMDMs were pretreated with Ca-074Me or with the cathepsin L inhibitor CAA 0225, primed with LPS and stimulated with silica (250 μg/ml, 6 h) or with FliDot (1 μg/ml, 6 h). The bars represent the average of two independent experiments performed in technical triplicates ± SD. *p < 0.05 **p < 0.01; ****p < 0.0001 (Two-way ANOVA) when compared to untreated cells. **H** PMA-differentiated (200 ng/mL, 24 h) shRNA-scramble and shRNA-CTSB THP-1 cells were transfected with ultrapure flagellin from *S. typhimurium* using Lipofectamine 3000 (1 μg/ml, 4 h). IL-1β secretion was assessed in the culture supernatant by ELISA. The numbers represent the mean ± SD of experimental triplicates **p < 0.01, (Two-way ANOVA) when compared to untreated cells. Representative data from two independent experiments.

In order to evaluate the participation of cathepsins in the regulation of NAIP/NLRC4-mediated responses, murine macrophages were stimulated with ultrapure flagellin from *Salmonella typhimurium* in its free form (Fli) or inserted into lipid vesicles (FliDot), which improves the delivery of the agonist into the cellular cytosol [27]. Fli (Fig. 1A) or FliDot, but not empty vesicle (DOTAP), induced mature IL-1β release and caspase-1 processing by macrophages from C57BL/6 wild-type (WT) mice (Fig. 1A, B and Supplemental Material). Caspase-1 processing in response to cytosolic flagellin occurs independently of Toll-like Receptor (TLR)5 but requires NLRC4 (Supplemental Fig. 1A). Accordingly, the release of IL-1β (Fig. 1C) and lactate dehydrogenase (LDH) (Fig. 1D) was abrogated in *Naip1-7*^*−/−*^ and *Nlrc4*^*−/−*^ BMDMs, confirming the requirement of these molecules for the activation of inflammasome in response to cytosolic flagellin [18–20].

Of note, Ca-074Me prevented IL-1β secretion in response to cytosolic flagellin, a NAIP/NLRC4 trigger, in murine macrophages (Fig. 1E), and in a human monocytic cell line (phorbol 12-myristate 13-acetate (PMA)-differentiated THP-1 cells) (Fig. 1F), as previously described for NLRP3 agonists [28–32, 34]. Importantly, Ca-074Me was not cytotoxic and did not affect the cell death induced by inflammasome activation (Supplemental Fig. 1B) [35].

Since Ca-074Me is a presumed cathepsin B inhibitor but could also have inhibitory effects on other cathepsins such as L [30], we treated cells with CAA 0225, a specific cathepsin L inhibitor. Interestingly, CAA 0225 was able to reduce IL-1β release in response to silica, a NLRP3 agonist, but not in response to cytosolic flagellin (Fig. 1G), ruling out the participation of cathepsin L for NAIP/NLRC4 activation. To gain genetic evidence for the participation of cathepsin B in the responses to cytosolic flagellin, we took the advantage of lentivirus transduction in THP-1 cells in which its expression was significantly reduced by shRNA-CTSB (Supplemental Fig. 2A, B). As expected, IL-1β secretion by shRNA-CTSB cells in response to the nigericin, a classical NLRP3 agonist, was significantly lower than the control shRNA-scramble cells (Supplemental Fig. 2C), thus confirming the functional interference with the cathepsin B expression. Of note, IL-1β secretion in response to cytosolic flagellin was also significantly reduced in shRNA-CTSB cells (Fig. 1H), similar as observed for the effect of Ca-074Me on PMA-differentiated THP-1 cells (Fig. 1F), thus validating both pharmacological and genetic approaches to study the role of cathepsin B in inflammasome activation. Together, these findings indicate that, as described for NLRP3 inflammasomes, cathepsin B is also required for NAIP/NLRC4 activation in response to cytosolic flagellin.

### Lysosomal cathepsins modulate IL-1β production in response to flagellin independently of NLRP3 activation

**Fig. 2:**
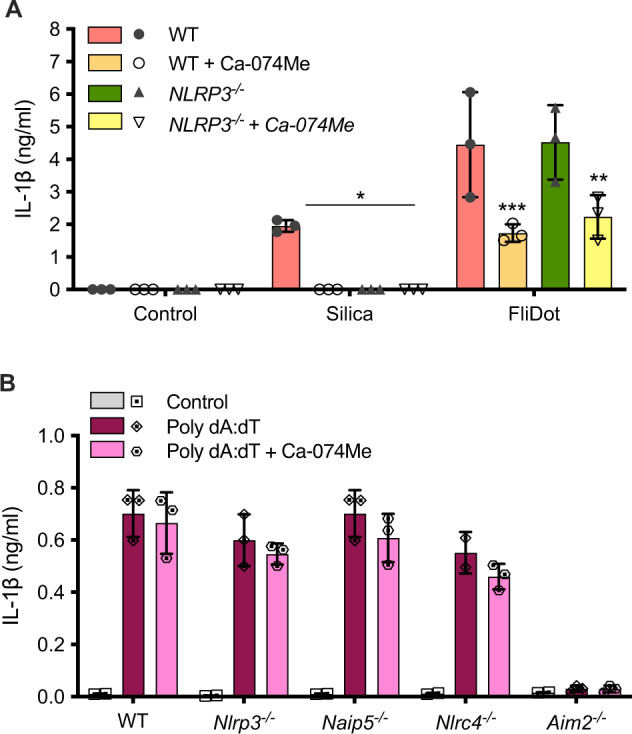
Lysosomal cathepsins modulate IL-1β production in response to flagellin independently of NLRP3 activation. Bone marrow-derived macrophages (BMDM) from C57BL/6j WT or *Nlrp3*^*−/−*^ mice were pretreated with the cathepsin B inhibitor Ca-074Me (25 μM), primed with LPS (200 ng/ml, 3 h). Next, the cells were stimulated with silica (250 μg/ml, 6 h) or with ultrapure flagellin extracted from *Salmonella typhimurium* inserted into DOTAP (FliDot) (1 μg/ml, 6 h). **A** IL-1β secretion was evaluated in the culture supernatants by HTRF. The bars represent the average of three independent experiments performed in technical triplicates ± SD. *p < 0.05; **p < 0.01; ***p < 0.001; ****p < 0.0001 (Three-way ANOVA) when compared to untreated cells. **B** Bone marrow-derived macrophages (BMDMs) from WT, *Nlrp3*^−/−^, *Naip5*^−/−^, *Nlrc4*^−/−^, and *Aim2*^−/−^ mice were pretreated with the cathepsin B inhibitor Ca-074Me, primed with LPS and transfected with poly dA: dT (1 μg/ml, 16 h). Secretion of IL-1β was determined by electrochemiluminescence in the culture supernatant. The bars represent the mean ± SD of experimental triplicates. Data representative of two independent experiments.

Since cathepsins are described as a common player in NLRP3 activation [28–31, 34, 36–38], we investigated whether cathepsins would modulate IL-1β release in response to flagellin through NLRP3. It is worthy of note that Ca-074Me diminished flagellin-induced IL-1β secretion in wild-type as well as in *Nlrp3*^−/−^ macrophages, indicating that cathepsins modulate NAIP/NLRC4-dependent IL-1β release in a NLRP3-independent manner (Fig. 2A). Interestingly, cathepsin inhibition had no effect on IL-1β secretion by WT, *Nlrp3*^−/−^, *Nlrc4*^−/−^ or *Naip5*^*−/−*^ macrophages in response to dsDNA (Poly dA: dT), a AIM2 agonist (Fig. 2B), indicating that cathepsins modulate NAIP/NLRC4 responses but are dispensable for AIM2 activation.

### Cathepsins play no role on the induction of pro-IL-1β during NAIP/NLRC4 inflammasomes activation

**Fig. 3:**
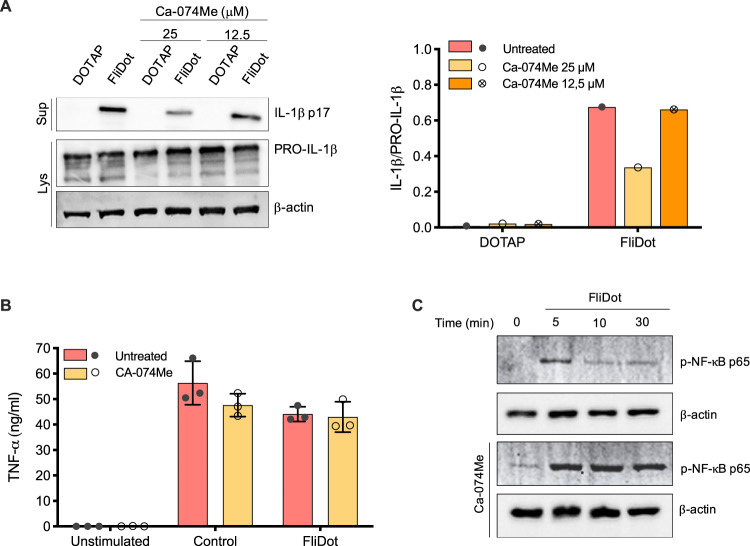
Cathepsins are dispensable for NF-kB-mediated responses related to the NAIP/NLRC4 priming step. **A** Starch-elicited peritoneal macrophages (PMs) isolated from C57BL/6 WT mice were treated with the cathepsin B inhibitor Ca-074Me (25 μM) for 1 h. Then, PMs were primed with LPS (200 ng/ml, 3 h) and incubated with empty DOTAP or with ultrapure flagellin extracted from *Salmonella typhimurium* inserted into DOTAP (FliDot) (1 μg/ml). The release of the active form of IL-1β was detected by western blot of the culture supernatant (Sup). Pro-IL-1β and β-actin were detected by western blot of the cellular lysates (Lys). Densitometry analysis was performed using ImageJ software and the relative expression of IL-1β was normalized by pro-IL-1β. Data representative of two-independent experiments. **B** Bone marrow-derived macrophages from C57BL/6j WT mice were pretreated with the cathepsin B inhibitor Ca-074Me, primed with LPS and stimulated with FliDot. TNF-α production was evaluated in the culture supernatants by HTRF. The bars represent the average of three independent experiments performed in technical triplicates ± SD. **C** PMs were pretreated with the cathepsin B inhibitor Ca-074Me for 1 h and stimulated with FliDot for the indicated periods. The phosphorylation of NF-κB were analysed by Western Blotting. β-actin served as reference protein. Data representative of three-independent experiments.

Orlowski et al. demonstrated that cathepsin B participates not only in the effector phase of NLRP3 activation but also contributes to the priming stage since its inhibition impairs pro-IL-1β transcription [30]. Therefore, we verified whether the inhibition of cathepsins could also interfere with the induction of pro-IL-1β in our experimental system. Ca-074Me clearly reduced the release of mature IL-1β in a dose-dependent manner (Fig. 3A and Supplemental Material) but had no effect on pro-caspase-1 (Supplemental Fig. 1C and Supplemental Material) and pro-IL-1β expression (Fig. 3A and Supplemental Material) in flagellin-stimulated macrophages. Accordingly, Ca-074Me treatment had no impact on TNF-α production (Fig. 3B), suggesting that cathepsins did not interfere with NF-κB-mediated gene transcription required for the induction of pro-IL-1β and TNF-α. In fact, the phosphorylation of NF-κB(p65) in response to cytosolic flagellin was even increased in the presence of Ca-074Me (Fig. 3C and Supplemental Material). These data suggest that, unlike what has been described for classical NLRP3 agonists, cathepsins are dispensable for the induction of pro-IL-1β during NAIP/NLRC4 inflammasomes activation by flagellin.

### Cathepsins are dispensable for ASC aggregation and caspase-1 cleavage

**Fig. 4:**
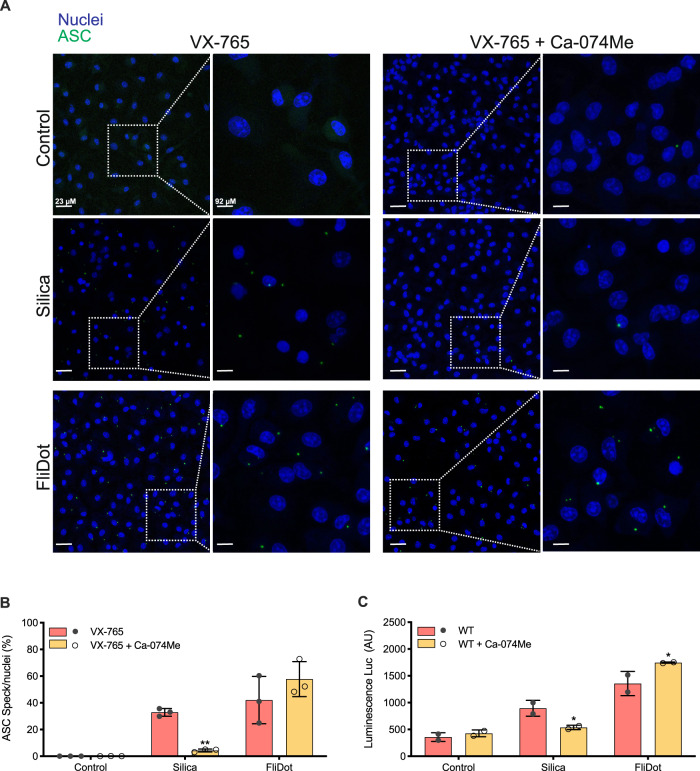
Cathepsins are not required to induce ASC aggregation into specks or caspase-1 cleavage. Bone marrow-derived macrophages (BMDM) from ASC mCitrine transgenic mice were pretreated with the cathepsin B inhibitor Ca-074Me and primed with LPS. Next, the cells were treated with the caspase-1 inhibitor VX-765 prior the stimulation with silica or with ultrapure flagellin extracted from *Salmonella typhimurium* inserted into DOTAP (FliDot). **A** Representative confocal imaging of cells showing the formation of ASC aggregates in their cytosol. ASC (green, mCitrine), nuclei (blue, DRAQ5). Data are representative of 3 independent experiments. **B** Quantification of ASC speck^+^ cells showed in **A**. The bars represent the average of three independent experiments ± SD. **p < 0.01 (Two-way ANOVA) **C**. Caspase-1 activity measured with Caspase-Glo®, arbitrary units (AU). The bars represent the average of two independent experiments ± SD. *p < 0.05 (Two-way ANOVA).

Inflammasome activation is related to the aggregation of the adaptor protein ASC into a complex known as the ASC speck [39]. Redistribution of cytosolic ASC to this complex has been widely used as a readout for inflammasome activation and precedes the downstream proteolytic cleavage of pro-IL-1β, pro-IL-18, and GSDMD [40]. The participation of cathepsins in the effector phase of NLRP3 inflammasomes activation has been extensively described [28–31, 34, 36–38]. To investigate whether cathepsins contribute to ASC speck formation in response to flagellin, macrophages from ASC-mCitrine transgenic mice [40] were treated with Ca-074Me. To prevent the release of ASC specks due to pyroptosis induction [41, 42], we blocked caspase-1 activity with VX-765 prior to inflammasome activation. As expected [34], the formation of ASC-mCitrine specks in response to silica was inhibited in the presence of Ca-074Me (Fig. 4A, B). However, the inhibition of cathepsins did not affect ASC speck aggregation in response to cytosolic flagellin, suggesting that cathepsins do not participate in NAIP/NLRC4 inflammasome assembly (Fig. 4A, B).

Of note, ASC specks in response to cytosolic flagellin were abrogated in *Nlrc4*^−/−^ and *Naip1-7*^−/−^ macrophages but not on *Nlrp3*^−/−^ cells (Supplemental Fig. 3A). In contrast, the ASC puncta in response to nigericin were similar among WT, *Nlrc4*^−/−^ and *Naip1-7*^−/−^ cells but they were lost on *Nlrp3*^−/−^ macrophages, thus demonstrating the specificity of the responses to the NAIP/NLRC4 and NLRP3 agonists (Supplemental Fig. 3B). Accordingly, Ca-074Me inhibited ASC specks aggregation in response to nigericin but not cytosolic flagellin.

In line with what was observed for ASC specking, cathepsin inhibition reduced caspase-1 activity in response to silica, but not in response to cytosolic flagellin, indicating that cathepsins do not contribute to caspase-1 cleavage (Fig. 4C and Supplemental Fig. 1C). Taken together, these data suggest that cathepsins act downstream of NAIP/NLRC4 inflammasome complex formation.

### Cathepsins and GSDMD act in a non-redundant manner to optimize IL-1β release in response to flagellin

**Fig. 5:**
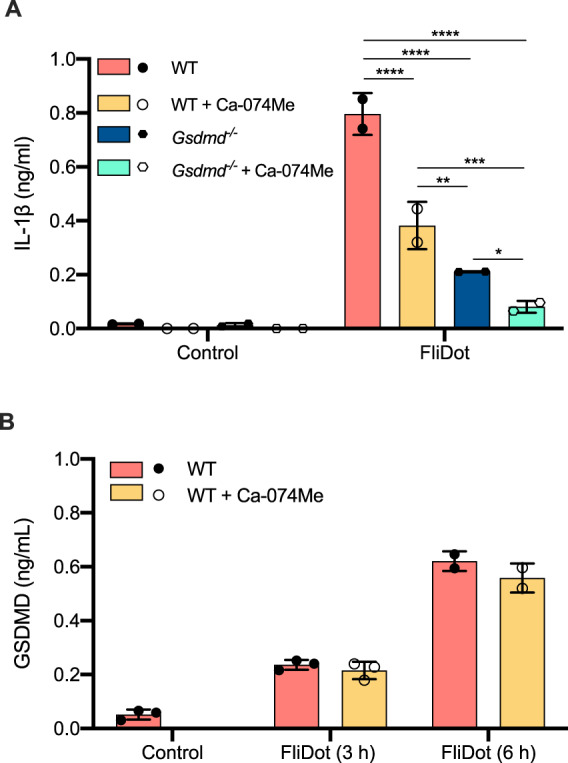
Ca-074Me and GSDMD have non-redundant roles on IL-1β release in response to flagellin. Starch-elicited peritoneal macrophages (PMs) isolated from C57BL/6 WT or *Gsdmd*^*−/−*^ mice were pretreated with the cathepsin B inhibitor Ca-074Me, primed with LPS and incubated with ultrapure flagellin extracted from *Salmonella typhimurium* inserted into DOTAP (FliDot). **A** IL-1β secretion was evaluated in the culture supernatants by ELISA after 3 h of stimulation. Data represent two experiments performed in technical triplicates ± SD. *p < 0.05; **p < 0.01; ***p < 0.001; ****p < 0.0001 (Three-way ANOVA). **B** GSDMD cleavage was determined in the culture supernatants by ELISA after 3 h and 6 h of stimulation. Data represent two (FliDot 6 h) or three (FliDot 3 h) independent experiments performed in technical triplicates ± SD.

**Fig. 6:**
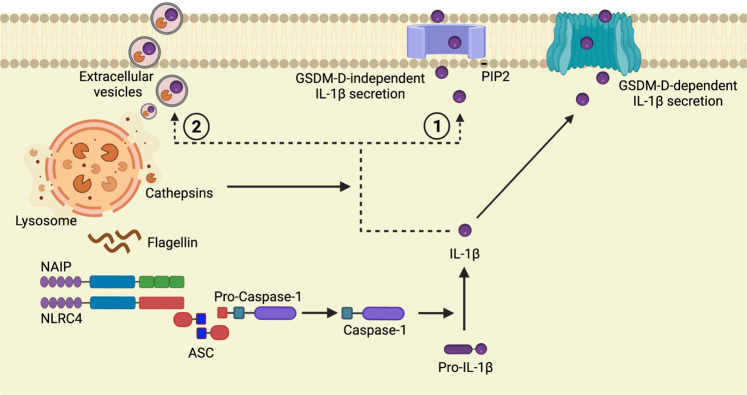
Proposed model for the role of cathepsin on NAIP/NLRC4-mediated IL-1β secretion. Flagellin stimulation leads to lysosome Membrane Permeabilization (LMP) resulting in the leakage of cathepsins into cell cytosol [27] where they participate in the regulation of NAIP/NLRC4-mediated responses. In contrast to that described for NLRP3 agonists [28–31, 34, 36–38], cathepsins did not interfere with the induction of pro-IL-1β, ASC speck formation or caspase-1 activity in response to flagellin, demonstrating their role downstream to NAIP/NLRC4 assembly. Here we propose the cooperation between cathepsins and GSDMD for the optimal IL-1β secretion by a mechanism that could involve mature IL-1β transportation to the plasma membrane [45] **(1)** or its release by extracellular vesicles [46–50] **(2)**.

IL-1β lacks a signal sequence peptide and follows an unconventional pathway for its secretion that is still not fully understood [43]. GSDMD mediates cell death by pyroptosis, and also contributes to IL-1β release through its transport via GSDMD pores, even by viable cells [44]. Thus, we investigated whether cathepsins would contribute to IL-1β release mediated by GSDMD. Pharmacological inhibition of cathepsins, as well as genetic ablation of *Gsdmd* partially reduced IL-1β release in response to flagellin (Fig. 5A). Importantly, IL-1β secretion in response to flagellin was abrogated in the absence of cathepsins and GSDMD (Fig. 5A). Curiously, the cleavage of GSDMD in response to cytosolic flagellin was not affected by the cathepsin inhibition (Fig. 5B). Our data support a cooperative role of cathepsins and GSDMD for NAIP/NLRC4-mediated IL-1β secretion (Fig. 6).

## Discussion

It is well-established in the literature that NAIP-NLRC4 inflammasome is activated through the direct binding of bacterial proteins [18–20]. In contrast, NLRP3 inflammasome is indirectly activated by cell disturbances caused by particulate material or pathogens. Increasing evidence in the literature demonstrate the participation of NLRC4 in inflammatory pathologies and non-bacterial infections [7–16]. The mechanisms involved in NAIP/NLRC4 activation under these conditions are still unknown, opening the possibility that endogenous signals could modulate NLRC4 activation in some circumstances, similarly to described for NLRP3. Here, we propose that lysosomal cathepsins boost NAIP/NLRC4-mediated mature IL-1β secretion independently of NLRP3.

The role of cathepsins in NLRP3 assembly and activation is extensively described [28–31, 34, 36–38]. In response to NLRP3 agonists, cathepsin inhibition blocked the formation of ASC specks and reduced caspase-1 activation [30, 34, 36]. Moreover, cathepsins also interfere with priming stage of NLRP3, leading to the pro-IL-1β transcription [30]. Here we demonstrate that cathepsins act in a distinct manner to regulate NAIP/NLRC4-mediated since the inhibition of cathepsins in flagellin-stimulated macrophages did not interfere with the induction of pro-IL-1β, ASC speck formation or caspase-1 activity, demonstrating their role downstream to NAIP/NLRC4 assembly. In line with these findings, IL-1β secretion in response to cytosolic flagellin was partially blocked in *Gsdmd*^−/−^ cells but was fully abrogated upon cathepsin inhibition, indicating that these two molecules act in concert to optimize its secretion.

Considering the predicted protease cleavage sites of pro-IL-1β provided by PROSPER server (https://prosper.erc.monash.edu.au/result3_queue.pl?id=91c5f4270c89d2eaca649ce11a6a0257-48), it is unlikely that cathepsins cleave IL-1β directly. We favor the idea that flagellin stimulation leads to LMP resulting in the leakage of cathepsins into cell cytosol [27] where they regulate alternative IL-1β secretion mechanisms (Fig. 6). Indeed, in addition to the GSDMD-dependent release of IL-1β, alternative/cooperative GSDMD-independent mechanisms for IL-1β secretion have been proposed. GSDMD-independent IL-1β secretion is slower [45] and can be used by viable cells [44] to maintain prolonged IL-1β secretion [45]. Such mechanisms include PIP2-mediated membrane transport [45] and release of extracellular vesicles [46–49] in which lysosomal proteins participate.

It has been recently reported that LAMP2A^+^ vesicles led to the secretion of low levels of IL-1β in LPS-treated monocytes [50]. The newly described caspase-1 target EEA1 is also required to induce a more robust IL-1β release independently of pyroptosis induction [51]. EEA1 is fundamental to induce early endosome endocytic membrane fusion and docking necessary to the traffic of compounds to other intracellular locations [52, 53]. Interestingly, cathepsin B localizes predominantly to early endosomes [54, 55], where it might have endopeptidase and exopeptidase activity. Importantly, the cleavage of pro-IL-1β change its isoelectric point since the pro-domain of IL-1β is negatively charged, whereas the mature p17 is positively charged. The positively charged mature IL-1β interacts with negatively charged phospholipid, such as PIP2, in the inner leaflet of the plasma membrane [45], thus facilitating its release through the GSDMD-dependent or—independent mechanisms. Although the exact mechanism involved in the cathepsin-mediated IL-1β secretion remains to be fully elucidated, these proteases could favor the maintenance of positively charged mature IL-1β and/or its traffic to the plasma membrane (Fig. 6).

Despite the controversies regarding the pathways that mediate IL-1β secretion, it is likely that the different mechanisms coexist to properly orchestrate the extravasation of this cytokine accordingly to stimulus intensity. Therefore, vesicular pathways or secretion directly through the plasma membrane appear to be important for mild stimuli. In contrast, GSDMD engagement and induction of pyroptotic cell death appear to be triggered in response to potent stimuli [56]. In addition, the divergent IL-1β externalization mechanisms observed in different studies may be related to the cell type, indicating that different cells may engage particular pathways for IL-1β release. Considering that cathepsin inhibition could impair IL-1β release by *Gsdmd*^−/−^ cells, it is fair to predict that those proteases mediate GSDMD-independent IL-1β secretion.

The studies on NAIP/NLRC4 inflammasome structure and activation revealed that additional regulatory mechanisms beyond ligand recognition could be required to boost its effector responses. In addition to the involvement of the cathepsin B described here, other non-canonical pathways for NAIP/NLRC4 activation include NLRP3 recruitment [13, 24, 25], NLRC4 ubiquitination by Sug1 [57] and phosphorylation by PKCδ [21, 22, 24] and LRRK2 [23]. Since reduced expression of PKCδ or LRRK2 results in defective but not ablated NAIP/NLRC4 activation [23, 24], it suggests that there are accessory and redundant mechanisms for releasing NLRC4 from the self-inhibiting state. These multiple regulatory pathways could be engaged during NAIP/NLRC4 activation in biological processes beyond bacterial infection, such as mucosal candidiasis [7], brain inflammation [15, 16], acute inflammatory hyperalgesia induced by carrageenan [12], hypertonic conditions [11], retinal degeneration [13], and obesity-related tumorigenesis [58].

Given the central role of inflammasomes in controlling infection and, also, induction of inflammatory pathologies, many efforts have been made to uncover novel molecules involved in their regulation. In this context, targeting cathepsins could be useful to regulate NAIP/NLRC4-induced IL-1β secretion as an alternative for drugs that block IL-1 signaling that are currently being used for the treatment of inflammasomes-driven immunopathologies but still present considered toxicity [59].

## Experimental procedures

### Mice

A total of 6–8 week-old wild type (WT) C57BL/6, *Tlr5*^−/−^, *caspase-1/11*^−/−^, *Nlrc4*^*−/−*^, *Nlrp3*^*−/−*^, *Aim2*^*−/−*^, *Naip1-7*^−/−^, *Gsdmd*^−/−^, and ASC-mCitrine transgenic mice were bred in our animal facilities at the Federal University of São Paulo, or at the animal facilities at the University Hospitals, Uni-Bonn. All animal studies were carried out in accordance with the Brazil ethical guidelines and have been approved by the local animal experimentation committee of the Federal University of São Paulo under the license 2015/ 9515131015.

### Preparation of mouse macrophages

For the generation of bone marrow-derived macrophages (BMDM), 8–12 week-old mice were euthanized, and cells isolated as described previously [60]. Briefly, bone marrow progenitor cells were recovered from the mouse femur and seeded in 75 cm^2^ flasks and incubated for full differentiation in DMEM (Thermo-Fischer) medium containing 20% L929-conditioned medium, 10% heat inactivated fetal bovine serum (FBS), 10 mM Hepes, 2 mM L-glutamine, and 100 units/mL streptomycin and penicillin (Invitrogen) for 6 days in a humidified incubator at 37 °C, under 5% CO_2_. For the isolation of peritoneal macrophages (PMs), 6- to 12-week-old mice were intra peritoneal injected with 2 ml of 1.5% starch solution from potatoes (Sigma-Aldrich). After 4 days, mice were euthanized and PMs were obtained by peritoneal lavage with ice-cold PBS 1×. Macrophages were cultured in RPMI medium supplemented with 3% FBS and antibiotics in a humidified incubator at 37 °C, under 5% CO_2_. After 3 h, non-adherent cells were removed by washing with warm PBS.

### Preparation of THP-1 cells

THP-1 cells (American Type Culture Collection) were cultured in RPMI 1640 (Life Technologies) supplemented with heat-inactivated 10% FBS (Life Technologies), 2 mM L-glutamine, 1 mM sodium pyruvate and 100 units/mL streptomycin and penicillin (Invitrogen) at 37 °C and 5% CO_2_. Cells were used until passage ten. THP-1 cells were differentiated into macrophage-like cells with 200 ng/ml PMA for 24 h followed by 24h-incubation with supplemented RPMI 1640.

### Macrophage stimulation with inflammasome agonists

PMs (5 × 10^5^) and BMDMs (1 × 10^5^) were primed for 3 h with LPS (200 ng/ml) (Invitrogen) in Opti-MEM for induction of immature forms of caspase-1 and IL-1β. After priming, cells were stimulated with inflammasome agonists. Ultra purified flagellin from *S. typhimurium* (1-3 μg/ml) (Invivogen) was inserted into N-[1-(2,3-dioleoyloxy)propyl]-N,N,N-trimethylammonium methyl-sulfate (DOTAP) (Roche Diagnostics), a cationic lipid formulation that permits delivery of proteins to the cytosol. DOTAP was used accordingly to the manufacturer’s instructions. Briefly, purified protein is added to DOTAP (5 μl of DOTAP to each 3 μg of protein) and incubated in the presence of Hepes-buffered saline. The mixture was gently homogenized for 1 minute and incubated at room temperature for 15 min. PMs and BMDMs were also stimulated with monosodium urate crystals (MSU) (250 μg/ml, 6 h) (Invitrogen) for 6 h, Silica (250 μg/ml, 6 h) or transfected with Poli dA:dT (1 μg/ml, 16 h) (Invitrogen). PMA-differentiated THP-1 cells were stimulated with ultra-purified flagellin from *S. typhimurium* (1–3 μg/ml) (Invivogen) transfected with lipofectamine 3000 (Invitrogen) for 4 h.

### Lentiviral Transduction and Immunofluorescence assay

For the generation of lentiviral vectors containing short-hairpin shRNA-pLKO CTSB, or shRNA-pLKO scramble sequences, co-transfection of 10 μg for each one (Sigma-Aldrich), vesicular stomatitis virus G protein expression plasmid (5 μg) and psPAX2 plasmid (carrying gag, pol, and rev genes) was performed using HEK 293 T packaging cell line, by a calcium phosphate protocol. After 48 h, the supernatant containing the retroviral particles was recovered and supplemented with 4 μg/mL polybrene and stored at –80 °C. These supernatants were used to transduce target undifferentiated THP-1 cells. The transduced cells were then selected with 10 µg/mL puromycin for two weeks. To evaluate the levels of cathepsin B expression in PMA differentiated shRNA-CTSB and shRNA-scramble cells, the immunofluorescence assay was performed using 5 × 10^4^ cells/well in 96-well plates. Cells were washed in PBS, fixed in 4% PFA in PBS, and incubated with anti-cathepsin B antibody (1:100; Santa Cruz) and with anti-rabbit AlexaFluor 488-conjugated (1:1000; Thermo-Fisher). The randomized fluorescent cells were acquired using the INCell Analyzer 2200™ (GE Healthcare) and fluorescence intensity was quantified by ImageJ software (NIH).

### Treatments

To assess the role of cathepsins in inflammasome-mediated responses, cells were cultured in the presence of the cathepsin B inhibitor Ca074-Me Me (N-(L-3-trans-propylcarbonyl-oxirane-2-carbonyl)-L-isoleucyl-L-proline methyl ester) (12.5–25 μM) (Enzo life sciences) or in the presence of the cathepsin L inhibitor CAA 0225 (25 μM) ((2S,3S)-oxirane-2,3-dicarboxylic acid 2-[((S)-1-benzylcarbamoyl-2-phenyl-ethyl)-amide] (Millipore). The inhibitors were added to the cell culture 1.5 h before priming and stimulation. To inhibit caspase-1, the inhibitor VX-765 (25 μM) was added in the last 10 min of LPS priming and before cell stimulation.

### Measurement of cytokine release

IL-1β was measured in culture supernatants by enzyme-linked immunosorbent assay (ELISA) kits from R&D Systems, by electrochemiluminescence kit from Meso Scale Discovery, or with Homogeneous Time Resolved Fluorescence (HTRF) kits from Cisbio following the manufacturer’s instructions. TNF-α release was measured with HTRF kits from Cisbio following the manufacturer’s instructions.

### Western blot

Electrophoresis of proteins was performed using a BIO-RAD Mini Protean Tetra System. Briefly, after stimuli cells supernatants were collected and the cells were washed once with ice-cold PBS 1×, lysed directly in SDS sample buffer (50 mM Tris pH 6,8, 320 mM β-mercaptoethanol, 2% SDS, 10% glycerol, 3 μM bromophenol blue), boiled for 5 min and analyzed for caspase-1, IL-1β, and phospho-NF-κB (p65). For caspase-1 and IL-1β release, the harvested supernatants were centrifugated for 10 min at 400 rcf, 4 °C. The supernatants were collected and precipitated with methanol/chloroform, as described previously [61]. The pellets were diluted at SDS sample buffer and boiled for 5 min. Samples were resolved under reducing conditions for 2 h at 120 V in SDS-polyacrylamide gels. Proteins were then transferred onto PVDF membranes for 1 h at 100 V in a wet system. Blots were blocked for 1 h in TBST (10 mM Tris-HCl, pH 7.4, 150 mM NaCl, and 0.05% Tween) containing 5% nonfat dried milk and then probed with polyclonal goat antibody to IL-1β (AF-401 R&D Systems) (1:500), with polyclonal rat antibody to caspase-1 (Genentech) (1:500) or with rabbit IgG to phospho-α-p-NF-κB (p65) (93H1 Cell Signaling) (1:1000). Reactions were detected with suitable secondary antibody (1:1000) conjugated to horseradish peroxidase (Santa Cruz Biotech) and visualized using an enhanced chemiluminescence solution (250 mM Luminol, 90 mM p-Coumaric Acid, 1 M Tris/HCL pH 8.5, 30% H_2_O_2_).

### Cell cytotoxicity/viability assays

Cell cytotoxicity was evaluated by the lactate dehydrogenase (LDH) activity that was released from permeable cells. Supernatants were collected after 45 min up to 3 h of stimulation and LDH release was measured with a Cytotoxicity Detection Kit^plus^ (Roche), accordingly to the manufacturer’s instructions. Cell viability was analyzed using the Cell Titer-Blue (CTB) Cell Viability Assay (Promega) as indicated by the manufacturer.

### ASC speck analysis

BMDMs from ASC-mCitrine transgenic mice were fixed with 4% formaldehyde and nucleic acids were stained with 2.5 µM DRAQ5 (eBioscience). Cells were imaged using an Observer.Z1 epifluorescence microscope, 20× objective (dry, PlanApochromat, NA 0.8; ZEISS), Axiocam 506 mono, and ZEN Blue software (ZEISS), followed by analysis with Cell profile software. For representative images, a Leica TCS SP5 SMD confocal system (Leica Microsystems) was used for confocal laser-scanning microscopy. Images were acquired with a 63X objective, followed by analysis with Fiji software. For ASC speck analysis on *Nlrc4*^*−/−*^, *Nlrp3*^*−/−*^, and *Naip1-7*^*−/−*^ macrophages, cells were plated overnight in a 96-well black plate (Greiner) with a clear bottom for microscopy. On the next day, cells were stimulated with cytosolic flagellin or nigericin for indicated times and were fixed with 4% paraformaldehyde (Sigma Aldrich) for 20 min. After washes, cells were permeabilized with buffer containing 10% BSA (Sigma Aldrich), 1% FBS (LGC), 0.5% Triton-X100 (Sigma Aldrich), diluted in PBS for 1 h at room temperature. Wells were washed twice with warm PBS and incubated overnight at 4 °C with 1:1000 anti-ASC (Millipore, clone 2EI-7) followed by the incubation with secondary antibody Alexa-fluor 647 (Invitrogen) 1:1000 for 1 h at room temperature. Then, cells were incubated with DAPI 5 mg/mL (Sigma Aldrich) and images were acquired on IN Cell Analyzer 2200.

### Statistical analysis

The sample sizes required for the experiments were estimated based on the preliminary results. No blinding or randomization was performed in any of the experiments. Statistical analysis was performed with GraphPad Prism 8.0 software (San Diego). All data were expressed as means ± standard deviation (SD). Statistical significance was determined by the three-way, two-way, or one-way ANOVA followed by Tukey post hoc test as indicated in figure legends. Alternatively, Student’s t-test was used when two groups with a single variable were compared. Data were considered significant when *p* ⩽ 0.05 (^∗^), 0.01 (^∗∗^), 0.001 (^∗∗∗^), or 0.0001 (^∗∗∗∗^).

## Supplementary information

aj checklist

Supplementary Figure Legends

New Supplemental Figure 1

New Supplemental Figure 2

New Supplemental Figure 3

Supplemental material

## Data Availability

All data generated or analyzed during this study are included in this published article [and its supplementary information files].
