# Supplementary material for: Lysosomal cathepsins act in concert with Gasdermin-D during NAIP/NLRC4-dependent IL-1β secretion
Source: Cell Death Dis. 2022 Dec 8;13(12):1029. doi: 10.1038/s41419-022-05476-3 (PMC9731969; doi:10.1038/s41419-022-05476-3)
Supplement: Supplementary file 2 — Supplementary Figure Legends [file 41419_2022_5476_MOESM2_ESM.docx]

**Supplemental Figure 1**. **(A)** Starch-elicited peritoneal macrophages (PMs) isolated from C57BL/6 WT, *Tlr5^-/-^* and *Nlrc4^-/-^* mice were primed with LPS and incubated with empty DOTAP or with ultrapure flagellin extracted from *Salmonella typhimurium* inserted into DOTAP (FliDot). The presence of the active form of caspase-1 was detected by western blot of the culture supernatant. Data represent two independent experiments. **(B)** Cell viability assay (CTB) of WT BMDMs pretreated with the cathepsin B inhibitor Ca-074Me, primed with LPS. and stimulated with FliDot. The bars represent the average of three independent experiments performed in technical triplicates ± SD. **(C)** PMs isolated from C57BL/6 WT were treated with the cathepsin B inhibitor Ca-074Me. Then, cells were primed with LPS and incubated DOTAP or FliDot. The presence of the active form of caspase-1 was detected by western blot in the culture supernatant (Sup). Pro-caspase-1 were detected by western blot of the cellular extracts (Lys). Data represent two independent experiments.

**Supplemental Figure 2.** **(A)** Representative fluorescence images of cells showing the expression of cathepsin B in their cytosol (green - Cathepsin B; blue - nuclei (DAPI)). **(B)** Cathepsin B mean fluorescence intensity (MFI) was measured in shRNA-scramble and shRNA-CTSB THP-1 cells. The bars represent MFI for twenty-four regions of interest (ROIs) among six different fields for each cell line ± SD, **p<0.01, (Student’s t test). **(C)** PMA-differentiated (200ng/mL, 24h) shRNA-scramble and shRNA-CTSB THP-1 cells were treated with nigericin (10 μM, 4h). IL-1β secretion was assessed in the culture supernatant by ELISA. The bars represent the mean ± SD of experimental triplicates ***p<0.0001, (Student’s t test). Data representative of two independent experiments.

**Supplemental Figure 3**. Bone marrow-derived macrophages (BMDM) from WT, *Nlrp3*^-/-^, *Naip1-7*^-/-^ and *Nlrc4*^-/-^ were pretreated with cathepsin inhibitor Ca-074Me for 1.5 h and primed with LPS (50 ng/mL) for 3 h at 37ºC and 5% CO_2_. Next, cells were incubated with (**A**) 3 μg/mL of ultrapure flagellin extracted from *Salmonella* Typhimurium inserted into DOTAP vesicle for 1,5 h (FliDot) or (**B**) 10 μM of Nigericin for 1 h. Cells were fixed, blocked, permeabilized and incubated with specific antibodies. Data were obtained on IN Cell Analyzer 2200 and are representative of at least two independent experiments. ***p<0.001; ****p<0.0001 ± SD.
