# Supplementary figures and images for: Lysosomal cathepsins act in concert with Gasdermin-D during NAIP/NLRC4-dependent IL-1β secretion
Source: Cell Death Dis. 2022 Dec 8;13(12):1029. doi: 10.1038/s41419-022-05476-3 (PMC9731969; doi:10.1038/s41419-022-05476-3)

**A**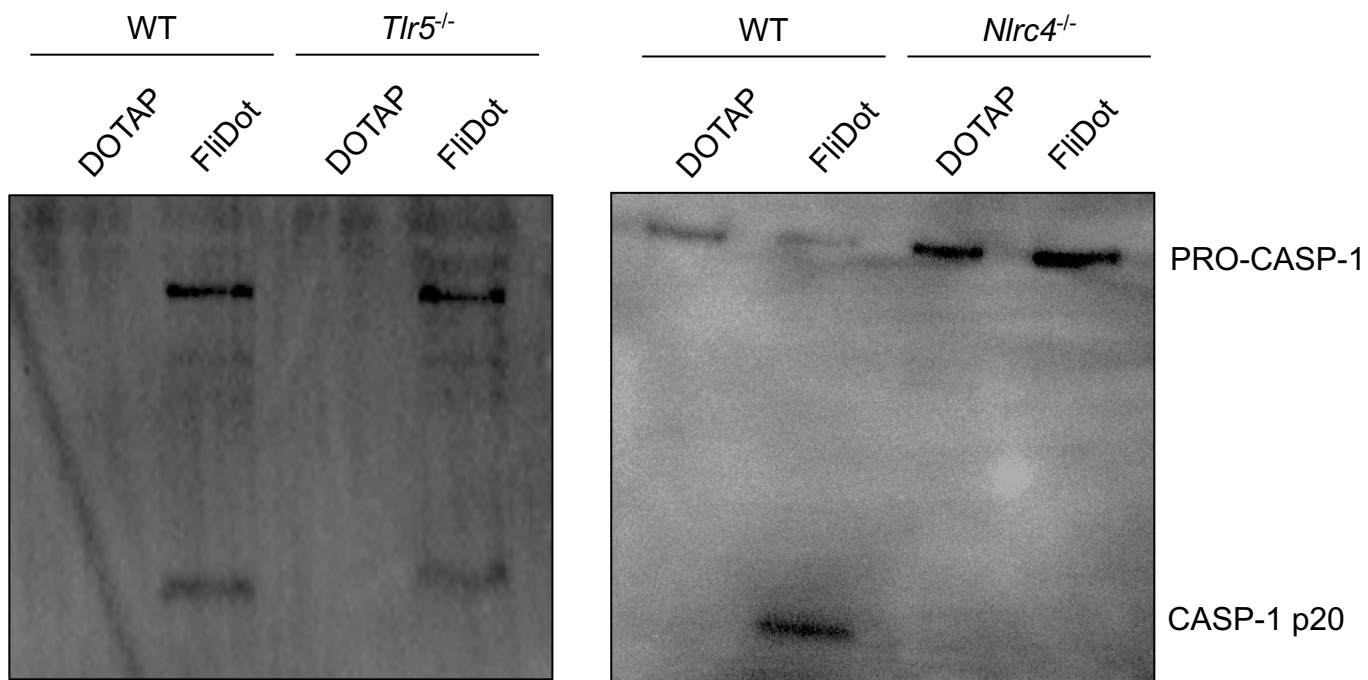**B**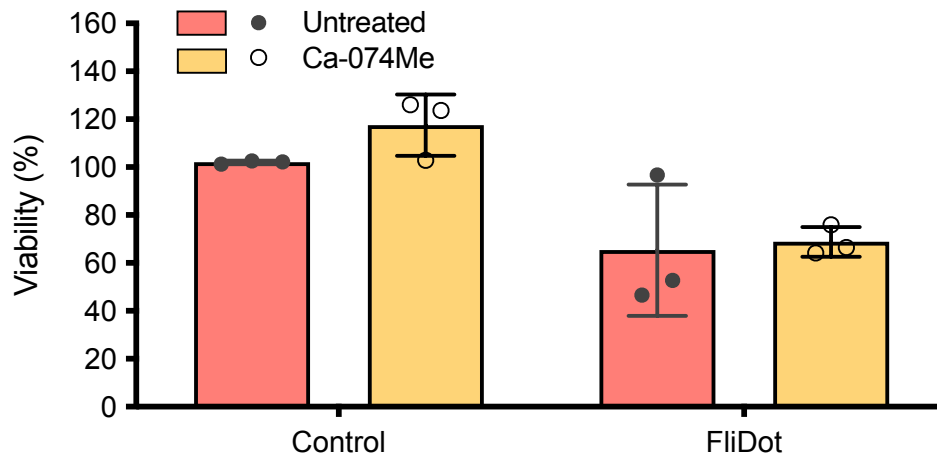**C**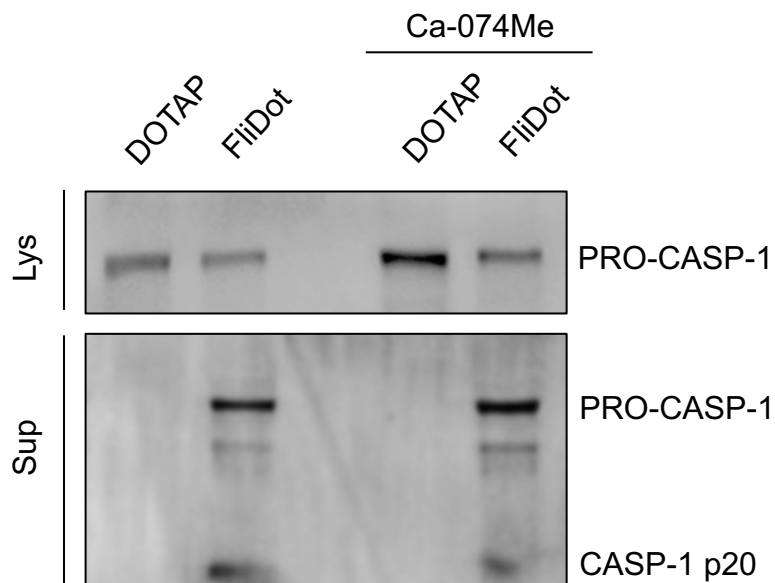

Supplement: Supplementary file 3 — New Supplemental Figure 1 [file 41419_2022_5476_MOESM3_ESM.pdf]

**A**

shRNA-scramble

shRNA-CTSB

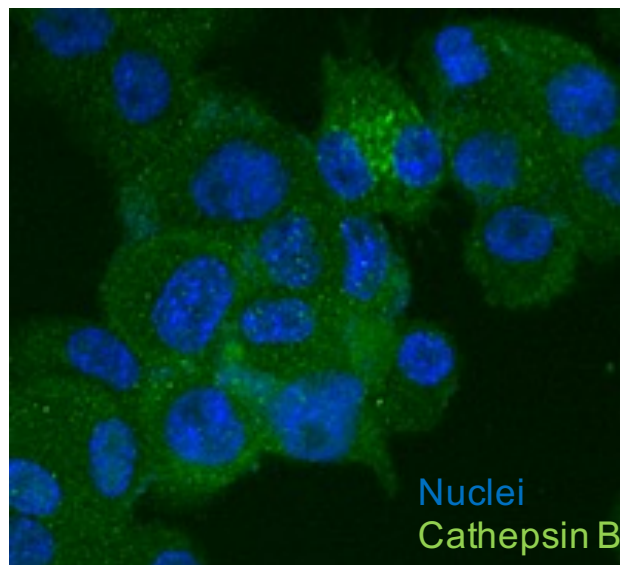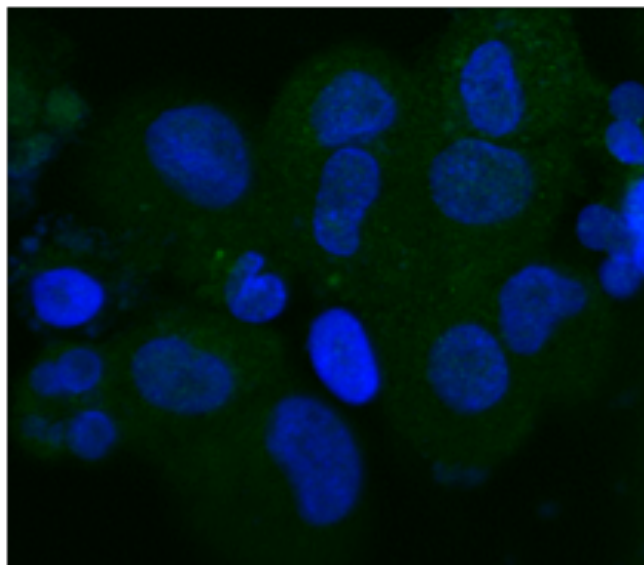**B**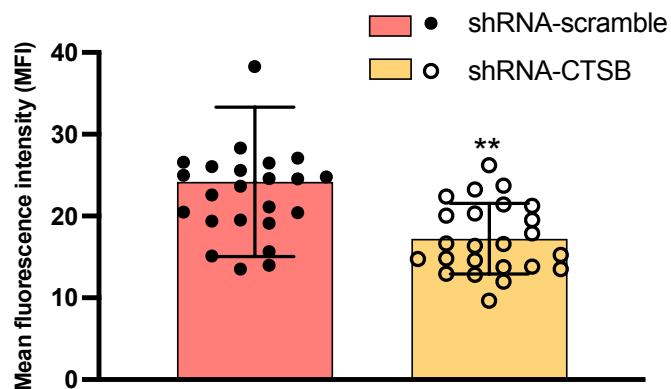**C**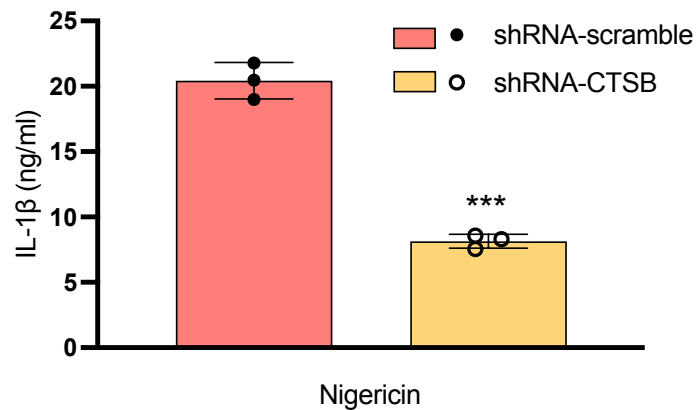

Supplement: Supplementary file 4 — New Supplemental Figure 2 [file 41419_2022_5476_MOESM4_ESM.pdf]

A

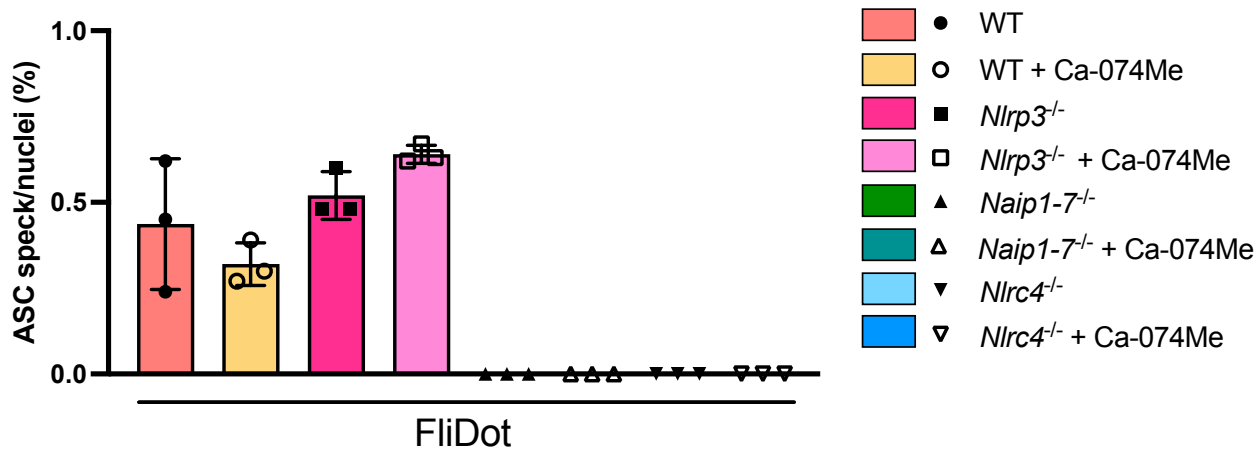

B

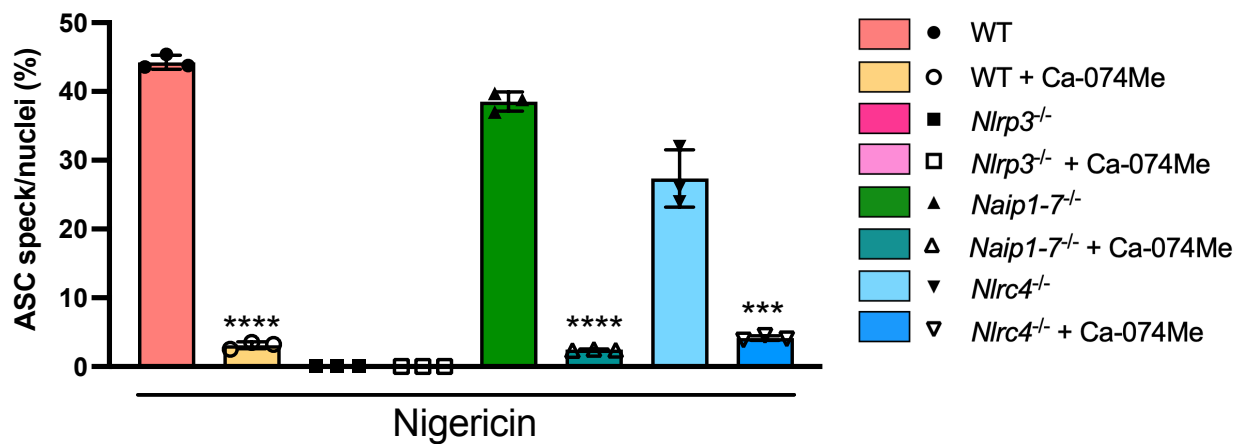

Supplement: Supplementary file 5 — New Supplemental Figure 3 [file 41419_2022_5476_MOESM5_ESM.pdf]

**Figure 1B**

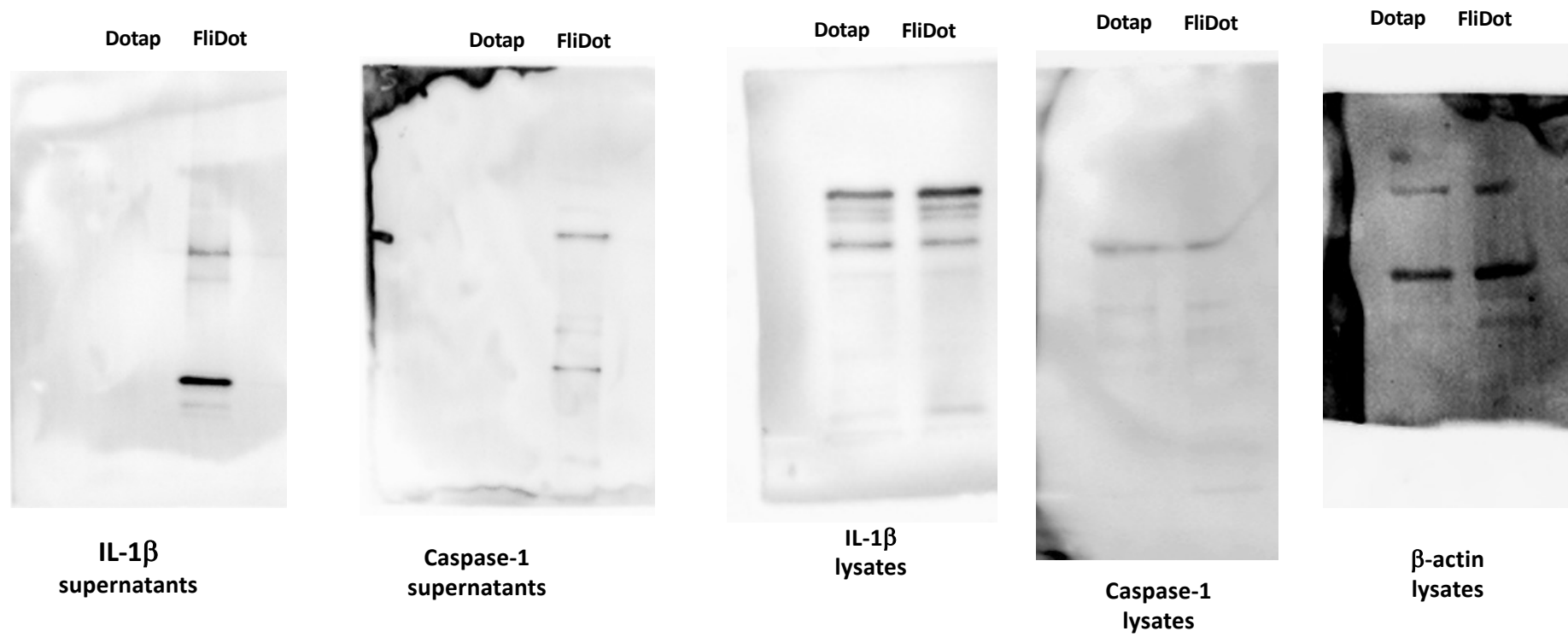

Figure 3A

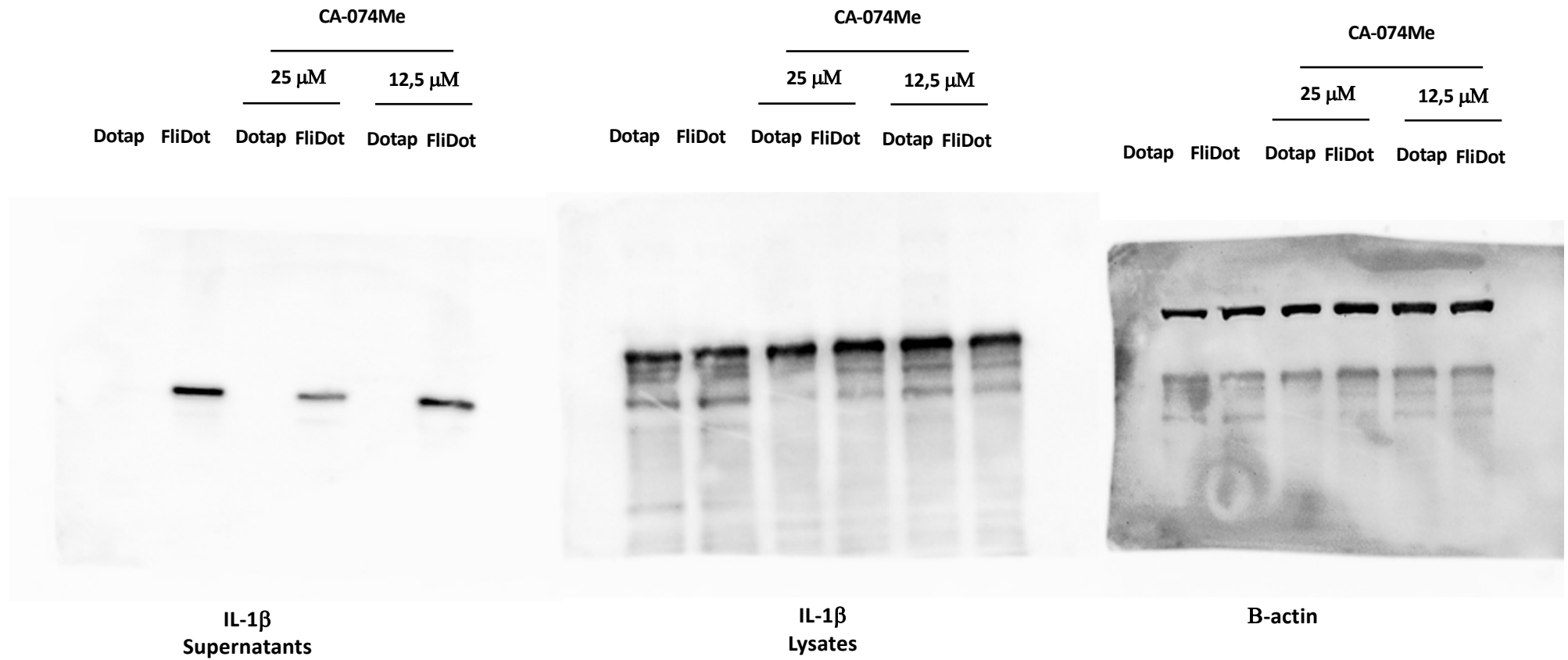

Figure 3C

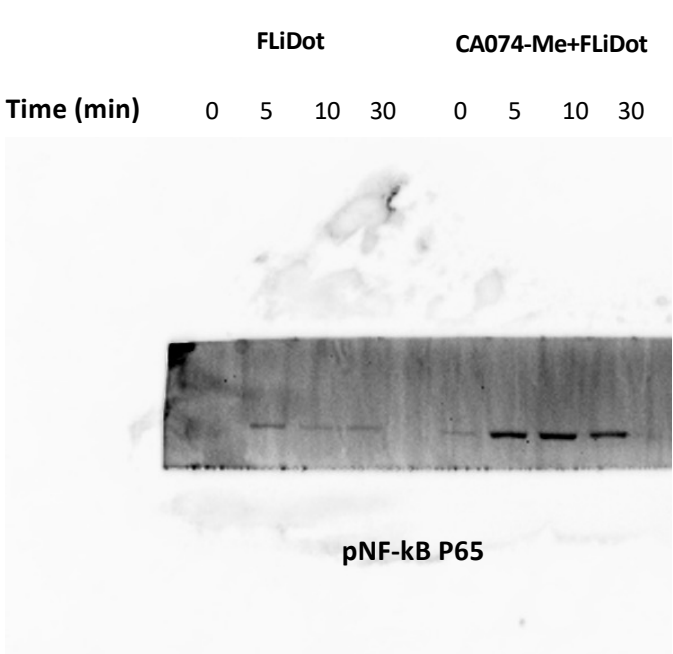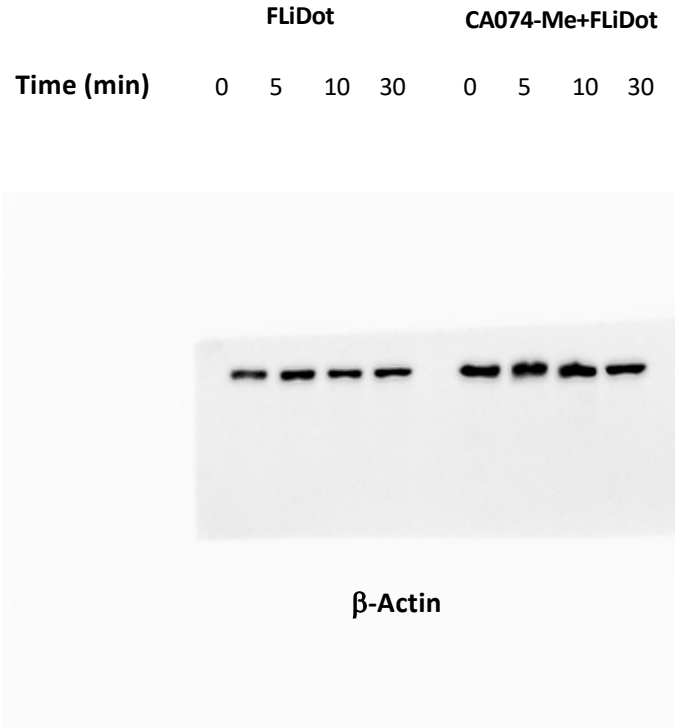

**SUPPLEMENTAL Fig 1B**

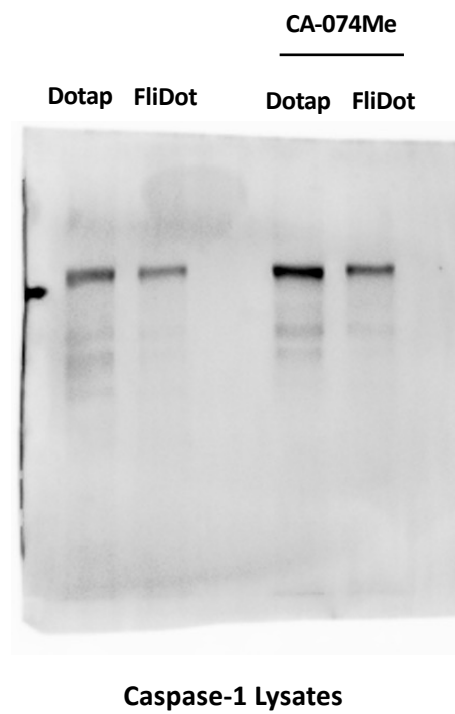

Supplement: Supplementary file 6 — Supplemental material [file 41419_2022_5476_MOESM6_ESM.pdf]
